# Muscle quality index correlates with arthritis: a cross-sectional study from NHANES 2011–2014

**DOI:** 10.3389/fmed.2025.1573729

**Published:** 2025-05-06

**Authors:** Chao Wang, Haotai Xu, Lei Wang, Sidan Huang, Bingcheng Pan, Hui Pan, Haisheng Jia, Shujie Tang

**Affiliations:** ^1^School of Traditional Chinese Medicine, Jinan University, Guangzhou, China; ^2^Zhongshan Hospital of Traditional Chinese Medicine Affiliated to Guangzhou University of Traditional Chinese Medicine, Zhongshan, China; ^3^School of Acupuncture-Moxibustion and Tuina, Hebei University of Chinese Medicine, Shijiazhuang, China; ^4^Affiliated Jiangmen Traditional Chinese Medicine Hospital of Jinan University, Jiangmen, Guangdong, China

**Keywords:** muscle quality index, arthritis, NHANES, public health, cross-sectional research

## Abstract

**Objectives:**

This study aimed to investigate the association between Muscle Quality Index (MQI) and arthritis using data from the National Health and Nutrition Examination Survey (NHANES) 2011–2014.

**Methods:**

A cross-sectional analysis was conducted using data from 4,558 participants aged 20–60 years. MQI was calculated using handgrip strength and appendicular skeletal muscle mass obtained through Dual-energy X-ray Absorptiometry (DXA) scans. Arthritis status was determined based on self-reported physician diagnoses. Multivariable logistic regression models were applied to examine the relationship between MQI and arthritis, adjusting for potential confounders, including demographic, socioeconomic, and lifestyle factors. Subgroup and sensitivity analyses were performed to evaluate heterogeneity.

**Results:**

Higher MQI was significantly associated with a lower risk of arthritis (adjusted OR = 0.73, 95% CI: 0.61–0.88, *P* = 0.001). Subgroup analyses revealed stronger associations in participants aged 30–40 years (OR = 0.47, 95% CI: 0.31–0.71, *P* < 0.001), females (OR = 0.68, 95% CI: 0.54–0.87, *P* = 0.002), and non-hispanic black people individuals (OR = 0.62, 95% CI: 0.43–0.89, *P* < 0.01). Sensitivity analysis confirmed a dose-response relationship, with participants in the highest MQI quartile having significantly lower odds of arthritis compared to the lowest quartile (adjusted OR = 0.69, 95% CI: 0.50–0.96, *P* = 0.027).

**Conclusions:**

MQI is inversely associated with arthritis prevalence, suggesting that maintaining muscle quality may be a critical factor in arthritis prevention. These findings highlight the importance of muscle health in public health strategies for arthritis management.

## 1 Introduction

Arthritis is a group of diseases involving inflammation and degeneration of the joints and is a leading cause of pain, disability, and reduced quality of life worldwide (1). Among them, osteoarthritis (OA) and rheumatoid arthritis (RA) are the most common types in clinical practice, in addition to more than 100 different subtypes of the disease with other etiologies and clinical manifestations (2). Osteoarthritis (OA) is estimated to affect more than 240 million people worldwide, including more than 32 million in the United States (3). RA is estimated to affect ~24.5 million people worldwide, with 25 to 50 new cases per 100,000 people each year (4). Due to its high prevalence and chronic nature, RA represents a significant economic burden to healthcare systems worldwide (5, 6).

The muscle mass index (MQI), proposed by Janssen et al. (7) in 2002, assesses an individual's muscle mass by combining variables such as an individual's weight and height to measure an individual's muscle mass accurately. Compared to the traditional Body Mass Index (BMI), the MQI better reflects the ratio of muscle to fat and avoids misclassification that can occur when relying on weight alone for assessment (8). In addition, the MQI has the advantage of being a simple measurement that provides accurate information about an individual's muscle health (9). In recent years, the MQI has been used extensively in several studies, and using data from the National Health and Nutrition Examination Survey (NHANES), researchers have examined the relationship between the MQI and a variety of health indicators. Several studies have shown that a low MQI is strongly associated with a variety of health problems, such as decreased mobility, increased risk of falls, and increased hospitalization rates (10). In addition, MQI has been associated with respiratory health, cardiovascular disease, and sleep (11–13).

Although several studies have investigated the association of MQI with a number of chronic diseases (13, 14), the potential relationship between MQI and arthritis remains understudied. In recent years, the MQI has been shown to be negatively associated with several indicators of obesity, particularly abdominal obesity (14, 15). A study by Baah et al. (16) showed a significant association between arthritis and obesity, sedentary behavior, and aging. We wondered whether MQI might affect bone and joint health, so we conducted the present study using data from the National Health and Nutrition Examination Survey (NHANES) to examine the relationship between muscle mass index (MQI) and arthritis [including rheumatoid arthritis (RA), osteoarthritis (OA), and psoriatic arthritis (PSA)] in the U.S. population aged 19 years to < 60 years. The relationship between the MQI and arthritis [including rheumatoid arthritis (RA), osteoarthritis (OA), and psoriatic arthritis (PSA)] was rigorously adjusted for key covariates using diverse and broadly representative data sources.

## 2 Methods

### 2.1 Study participants

**Figure 1:**
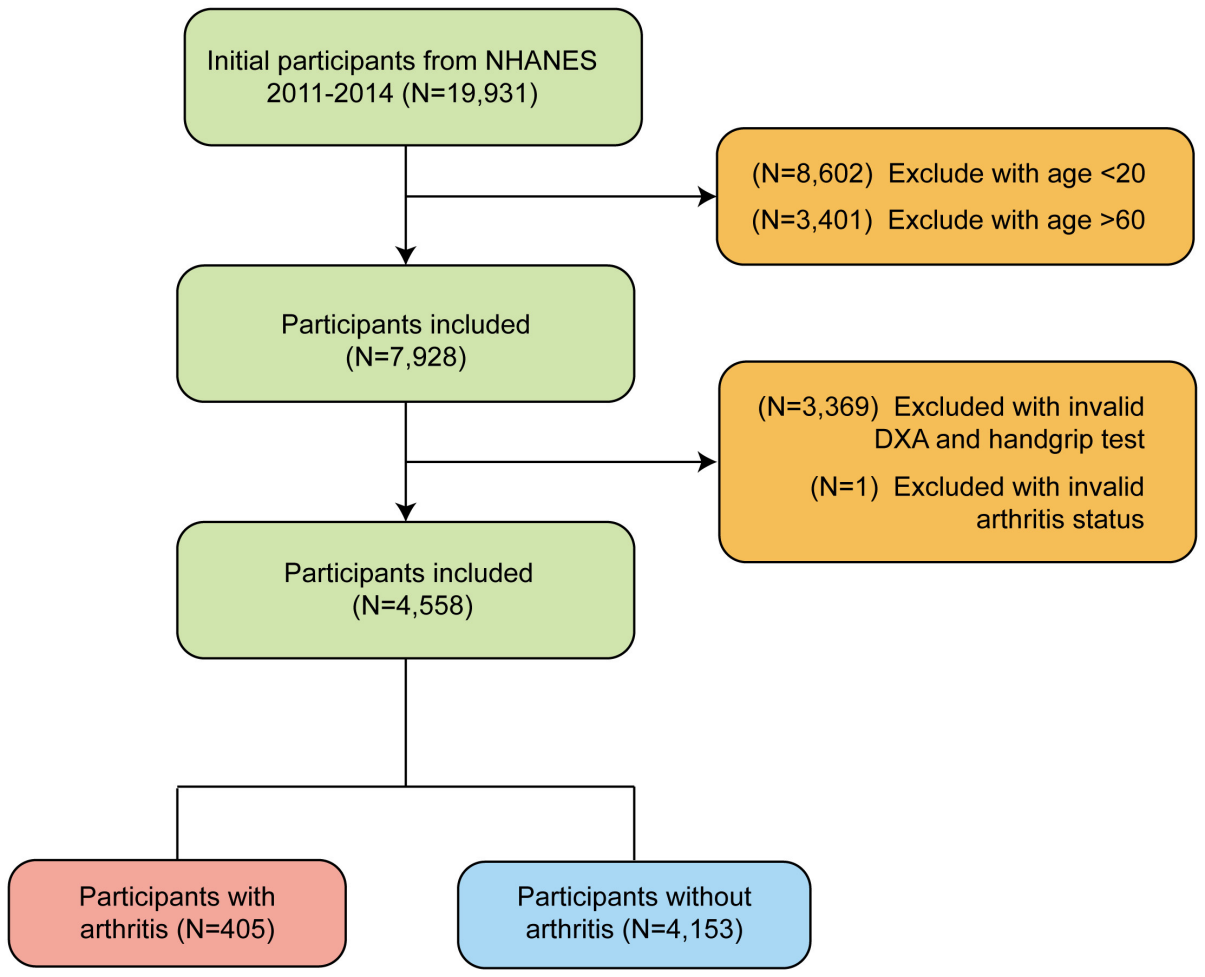
Flow chart of participants selection of participants from NHANES 2011–2014 based on MQI (Muscle Quality Index).

This study used data from NHANES between 2011–2014, totaling 19,931 participants. Participants younger than 20 years and older than 60 years were first excluded to meet the study criteria (*N* = 8,602). A final total of 7,928 participants were included. Next, we further excluded 3,369 participants who were missing valid DXA scan data and grip strength testing and 1 participant who was missing information on arthritis status. Ultimately, a total of 4,558 eligible study participants were enrolled, including 405 participants with arthritis and 4,153 participants without arthritis. The flow of the study design is shown in Figure 1.

### 2.2 Exposure and outcome definitions

For participants' MQI, which was assessed by standardized measurement calculations of laboratory data, MQI was defined as the ratio of the joint grip strength value divided by Appendicular skeletal muscle mass (ASM). Grip strength (HGS) is considered one of the measures of muscle strength throughout the body, and the joint grip strength value is the sum of the highest grip strength values of the extremities. To participate, test subjects needed to have not undergone surgery within the past 3 months and not have hand or wrist pain. During the test, participants stood and were asked to grip the dynamometer as tightly as they could. Three tests were performed on each hand at 60-second intervals, and the highest grip strength value for each hand was recorded. The ASM was obtained by DXA scanning, and ASM comprises lean mass (i.e., non-fat and non-bony tissue) of the arms and legs (11, 17). Arthritis, on the other hand, was assessed by arthritis-related questions in a medical condition questionnaire, in which participants answered the question: has a doctor or other health professional told {you/SP} {you/she/he}...have arthritis? If the participant answered “yes” to the first question, the participant was identified as an individual with arthritis, otherwise a non-arthritic individual. Although the NHANES data collection process is systematic and accurate, the reliance on self-reported data introduces the potential for recall bias.

### 2.3 Study variables

In this study, to control for confounding effects, we included a range of covariates in the analysis. Included were age, sex, race, marital status, education level, smoking, alcohol consumption, diabetes status, sedentary time, sleep duration, and vitamin B12 intake. Racial categories included Mexican American, other Hispanic/other race, non-hispanic white people, and non-hispanic black people. Marital status was categorized as “married or living with a partner,” “divorced/widowed/separated,” and “never married.” Educational level was categorized as “less than high school,” “high school,” and “college and above. Participants were categorized into “people with diabetes” and “people without diabetes” based on their diabetes status, which was defined by the participant's response to the question, “Have you ever been told by a doctor that you have these diseases?” If they answered “yes,” they were considered to have the disease. Three populations were categorized based on smoking history, with the “never smokers” defined as those who had smoked fewer than 100 cigarettes in their lifetime. This usually includes people who have never smoked or people who have tried but smoked very little. “Former smokers” are people who have smoked more than 100 cigarettes in their lifetime but no longer smoke at the time of the survey. This means that the respondent used to smoke but had quit at the time of the survey. “Current smokers” are those who have smoked more than 100 cigarettes in their lifetime and are still smoking. This includes occasional smokers (e.g., weekly or monthly smokers) and daily smokers. Alcohol drinkers were categorized as “never drinkers,” “moderate drinkers,” and “heavy drinkers,” also based on questions about alcohol use from the NHANES questionnaire. Vitamin B12, sedentary time, and sleep duration were continuous variables measured in pg/ml, minutes, and hours, respectively, to measure the level of vitamin intake, daily sedentary time, and sleep duration, and are expressed as mean ± standard deviation.

### 2.4 Statistical analysis

All statistical analyses in this study were performed following the Centers for Disease Control and Prevention (CDC) guidelines. Continuous variables were expressed as means and standard deviations, and categorical variables were expressed as percentages. Analysis of variance (ANOVA) was used for normally distributed data, the Kruskal-Wallis test for asymmetrically distributed data, and the chi-square test for categorical variables to compare differences between groups. Multivariate logistic regression models were used to explore the independent relationship between the MQI index and OAB in the three different models. The ratio of ratios (OR) and their 95% confidence intervals (95% CI) were calculated. In Model 1, no adjustment was made for covariates. Model 2 was adjusted for gender, age, and race. Model 3 was adjusted for sex, age, race, marriage, educational level attainment, smoking, alcohol consumption, diabetes, sedentary time, sleep time, and vitamin B12 intake. The association between MQI and arthritis was further assessed using smooth curve fitting (penalized spline method) and generalized additive model (GAM) regression.

Regarding subgroup analyses, we subjected multiple covariates to stratified multifactorial regression analysis methods. In addition, a log-likelihood ratio test model was used to add an interaction term to test the heterogeneity of the association between subgroups. *P*-value < 0.05 was considered as a statistically significant difference. All analyses were performed using Empower software version 4.2 (http://www.empowerstats.com; X&Y Solutions, Inc., Boston, MA, USA) and R version 3.4.3 (http://www.Rproject.org, R Foundation).

## 3 Results

### 3.1 Characteristics of the study population

A total of 4,558 participants with a mean age of 38.04 years (±11.39) were included in this study, and there was a significant correlation between age and the level of MQI, with a higher mean age in the Q1 group (38.76 years) and a relatively lower mean age in the Q4 group (37.07 years) (*P* = 0.005). Gender also showed a correlation with MQI levels, with the proportion of males gradually increasing with increasing MQI, from 45.18% in the Q1 group to 59.91% in the Q4 group (*P* < 0.001). Regarding race, non-hispanic black people were associated with lower MQI levels, with the highest proportion of non-hispanic black people in the Q1 group (36.40%), while the proportion significantly decreased to 14.21% in the Q4 group (*P* < 0.001). In addition, the status of being married or living with a partner was significantly associated with higher levels of MQI, with the percentage of those married in the Q4 group at 62.37% (*P* < 0.001). In terms of economic status, the poverty income ratio (PIR) showed a negative correlation with MQI level, with the proportion of PIR less than 1 being the highest in the Q1 group (24.39%) and relatively low in the Q4 group (20.96%) (*P* = 0.001). Educational level was positively associated with higher MQI levels, with the highest proportion of those with a university degree or higher in the Q3 group (65.76%) and a slight decrease in the Q4 group (57.89%) (*P* = 0.004). Smoking history was also significantly associated with MQI levels, with the proportion of current smokers increasing with MQI, reaching 27.11% of current smokers in the Q4 group (*P* < 0.001). In terms of drinking behavior, higher MQI levels were associated with high alcohol intake, with the proportion of heavy drinkers in the Q4 group reaching 44.74% (*P* = 0.027). The prevalence of diabetes mellitus was significantly associated with lower MQI levels, with the highest prevalence of diabetes mellitus in the Q1 group (11.40%) and the lowest prevalence in the Q4 group (2.19%) (*P* < 0.001). Mean sleep duration was associated with MQI levels, with the Q1 group having a shorter mean sleep duration (6.67 h) compared to 6.84 h in the Q4 group (*P* = 0.001). In addition, arthritis was significantly associated with lower MQI levels, with a prevalence of arthritis of 11.93% in the Q1 group compared to 6.84% in the Q4 group (*P* < 0.001) (Table 1).

**Table 1:** Baseline characteristics of study population according to systemic muscle quality index tertiles quartile.

**Characteristics**	**Total (4,558)**	**MQI**				
		**Q1 (1,140)**	**Q2 (1,139)**	**Q3 (1,139)**	**Q4 (1,140)**	***P*** **-value**
**Age**	38.04 ± 11.39	38.76 ± 1.72	38.18 ± 1.61	38.17 ± 1.00	37.07 ± 1.17	0.005
**Gender**						< 0.001
Male	2,417 (53.03%)	515 (45.18%)	583 (51.19%)	636 (55.84%)	683 (59.91%)	
Female	2,141 (46.97%)	625 (54.82%)	556 (48.81%)	503 (44.16%)	457 (40.09%)	
**Race/ethnicity**						< 0.001
Mexican American	590 (12.94%)	135 (11.84%)	161 (14.14%)	136 (11.94%)	158 (13.86%)	
Other Hispanic/Other Race	1,226 (26.9%)	219 (19.21%)	300 (26.34%)	335 (29.41%)	372 (32.63%)	
Non-hispanic white people	1,720 (37.74%)	371 (32.54%)	431 (37.84%)	470 (41.26%)	448 (39.30%)	
Non-hispanic black people	1,002 (22.42%)	415 (36.40%)	247 (21.69%)	198 (17.38%)	162 (14.21%)	
**Marital status**						< 0.001
Never married	1,305 (28.63%)	381 (33.42%)	340 (29.85%)	295 (25.90%)	289 (25.35%)	
Married/living with partner	2,660 (58.36%)	587 (51.49%)	656 (57.59%)	706 (61.98%)	711 (62.37%)	
Widowed/divorced/ Separated	593 (13.01%)	172 (15.09%)	143 (12.55%)	138 (12.12%)	140 (12.28%)	
**PIR %(SE)**						0.001
< 1	997 (21.87%)	278 (24.39%)	239 (20.98%)	228 (20.02%)	239 (20.96%)	
(1,3)	1,841 (40.39%)	480 (42.11%)	465 (40.83%)	425 (37.31%)	475 (41.67%)	
≥3	1,720 (37.74%)	382 (33.51%)	435 (38.19%)	486 (42.67%)	426 (37.37%)	
**Education level**						0.004
Below high school	778 (17.07%)	187 (16.40%)	192 (16.86%)	171 (15.01%)	228 (20.00%)	
High school	955 (20.95%)	257 (22.54%)	227 (19.93%)	219 (19.23%)	252 (22.11%)	
College or above	2,825 (61.98%)	696 (61.05%)	720 (63.21%)	749 (65.76%)	660 (57.89%)	
**Smoking history**						< 0.001
Never smoke	2830 (62.09%)	738 (64.74%)	721 (63.30%)	710 (62.34%)	658 (57.72%)	
Former smoke	710 (15.58%)	159 (13.95%)	183 (16.07%)	195 (17.12%)	173 (15.18%)	
Current smoke	1,018 (22.33%)	243 (21.32%)	235 (20.63%)	234 (20.54%)	309 (27.11%)	
**Alcohol drinking**						0.027
No alcohol use	597 (12.44%)	175 (15.35%)	138 (12.12%)	126 (11.06%)	128 (11.23%)	
Moderate alcohol use	2,029 (44.52%)	484 (42.46%)	524 (46.01%)	519 (45.57%)	502 (44.04%)	
High alcohol use	1,962 (43.05%)	481 (42.19%)	477 (41.88%)	494 (43.37%)	510 (44.74%)	
**Diabetes mellitus**						< 0.001
Yes	271 (5.95%)	130 (11.40%)	74 (6.50%)	42 (3.69%)	25 (2.19%)	
No	42 (92.41%)	978 (85.79%)	1,047 (91.92%)	1,077 (94.56%)	1,110 (97.37%)	
Borderline	75 (1.65%)	32 (2.81%)	18 (1.58%)	20 (1.76%)	5 (0.44%)	
**Sendentary time (min/day)**						0.008
< 480	2,651 (58.16%)	629 (55.18%)	660 (57.95%)	654 (57.42%)	708 (62.11%)	
≥480	1,907 (41.84%)	511 (44.82%)	479 (42.05%)	485 (42.58%)	432 (37.89%)	
**Vitamin B12 (pg/ml)**	437.425 ± 367.076	425.95 ± 280.91	447.77 ± 480.48	434.38 ± 310.54	441.61 ± 364.57	0.082
Yes	405 (8.89%)	136 (11.93%)	118 (10.36%)	73 (6.41%)	78 (6.84%)	
No	4,153 (91.11%)	1,004 (88.07%)	1,021 (89.64%)	1,066 (93.59%)	1,062 (93.16%)	

MQI, muscle quality index; PIR, poverty income ratio.

In summary, significant associations between muscle mass index (MQI) and demographic characteristics and health-related factors were demonstrated. Higher MQI was strongly associated with a higher proportion of males, a higher proportion of non-hispanic white people, a higher proportion of married or cohabiting individuals, higher levels of educational attainment, lower rates of poverty income, and a lower prevalence of diabetes and arthritis. In addition, higher MQI was associated with less smoking behavior, longer sleep duration, and less sedentary behavior.

### 3.2 Association between muscle quality index and arthritis

Table 2 shows the results of the multivariate logistic regression analysis of the association between muscle mass index (MQI) and arthritis. In the model without adjustment for covariates (model 1), MQI was negatively associated with the prevalence of arthritis (OR = 0.62, 95% CI: 0.53–0.74, *P* < 0.001), i.e., each 1-unit increase in MQI was associated with a 38% reduction in the prevalence of arthritis. After adjusting for age, sex, and race (model 2), the association was attenuated (OR = 0.72, 95% CI: 0.60–0.87, *P* < 0.001); after further adjustment for the variables of education level, income-to-poverty ratio, marital status, diabetes mellitus, smoking status, drinking status, vitamin B12 level, sedentary activity, and hours of sleep (Model 3), the results remained consistent (OR = 0.73, 95% CI: 0.61–0.88, *P* = 0.001), i.e., for every 1-unit increase in MQI, the prevalence of arthritis decreased by 27%.

**Table 2:** Multivariate logistic regression to assess the association between MQI and arthritis (*N* = 4,558).

**Variable**	**Model 1**		**Model 2**		**Model 3**	
	**OR (95% CI)**	***P-value***	**OR (95% CI)**	***P-value***	**OR (95% CI)**	***P-value***
MQI	0.62 (0.53, 0.74)	< 0.001	0.72 (0.60, 0.87)	< 0.001	0.73 (0.61, 0.88)	0.001
Q1	Ref		Ref		Ref	
Q2	0.85 (0.66,1.11)	0.234	0.92 (0.70, 1.21)	0.553	0.97 (0.73, 1.30)	0.862
Q3	0.51 (0.38, 0.68)	< 0.001	0.56 (0.41, 0.77)	< 0.001	0.60 (0.43, 0.82)	0.002
Q4	0.54 (0.41, 0.73)	< 0.001	0.68 (0.50, 0.93)	0.016	0.69 (0.50, 0.96)	0.027
*P*-trend		< 0.001		0.001		0.003

In sensitivity analysis, MQI was converted from a continuous variable to a categorical variable (quartile).

Model 1 = No covariates were adjusted.

Model 2 = Age, gender, and race were adjusted.

Model 3 = Age, Gender, Race, Educational level, Income to poverty ratio (PRI), Marital status, Diabetes mellitus, Smoking status, Drinking status, Vitamin B12, Sedentary activity, Sleep duration were adjusted.

OR, odds ratio; 95% CI, 95% confdence interval; MQI, Muscle quality index.

In the sensitivity analysis, MQI was categorized by quartiles. Participants in the highest quartile (Q4) exhibited significantly lower arthritis prevalence in all models compared with the reference group (Q1). For example, in the fully adjusted model (Model 3), the ratio of Q4 was 0.69 (95% CI: 0.50-0.96, *P* = 0.027). The trend test showed a significant dose-response relationship (*P*-trend < 0.05 for all models). These results suggest that a higher MQI is independently associated with a lower prevalence of arthritis.

### 3.3 Subgroup analysis

**Figure 2:**
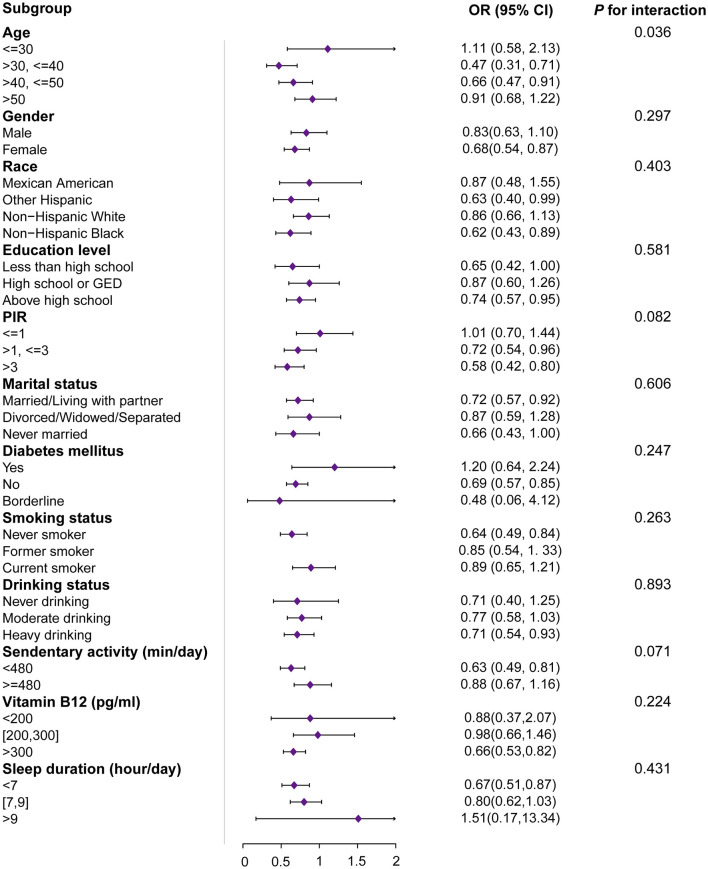
Subgroup and interactive analyses of MQI and arthritis from NHANES. OR, odds ratio; 95% CI, 95% confidence interval; MQI, muscle quality index; PIR, the ratio of family income to poverty. ^1^OR: odds ratio. ^2^95% CI: 95% confidence interval. The unit for continuous variables and the reference group for categorical variables are provided next to the variables. The OR of MQI was each unit increase in continuous variables and compared with the reference group for categorical variables.

Figure 2 demonstrates the subgroup analysis of the association between muscle mass index (MQI) and arthritis. Age subgroups showed significant heterogeneity (interaction *P*-value = 0.036). Higher MQI was significantly associated with a lower prevalence of arthritis in the 30 to 40-year-old group (OR = 0.47, 95% CI: 0.31–0.71, *P* < 0.001), and a similar protective effect was seen in the 40 to 50-year-old group (OR = 0.66, 95% CI: 0.47–0.91, *P* = 0.011). Sex-stratified analyses showed a significant negative association between MQI and arthritis prevalence in women (OR = 0.68, 95% CI: 0.54–0.87, *P* = 0.002) but not statistically significant in men (*P* = 0.194). Analyses by race showed that higher MQI was significantly associated with lower prevalence of arthritis among non-hispanic black people (OR = 0.62, *P* < 0.01) and other Hispanics (OR = 0.63, *P* = 0.047), whereas no significant associations were observed among non-hispanic white people and Mexican Americans. Education level and poverty-to-income ratio (PIR) also stratified the results, with higher socioeconomic status significantly associated with lower arthritis prevalence (e.g., PIR >3: OR = 0.58, 95% CI: 0.42–0.80, *P* = 0.001). Among the lifestyle factors, the prevalence of arthritis was significantly associated with a lower prevalence of arthritis in sedentary populations (daily sedentary activity time < 480 min) (OR = 0.63, 95% CI: 0.49–0.81, *P* < 0.001) and adequate vitamin B12 levels (>300 pg/ml: OR = 0.66, 95% CI: 0.53–0.82, *P* < 0.001) were significantly associated with arthritis prevalence. These results suggest that the relationship between MQI and arthritis prevalence is moderated by demographic, socioeconomic, and lifestyle factors.

### 3.4 Smoothing curve analysis

**Figure 3:**
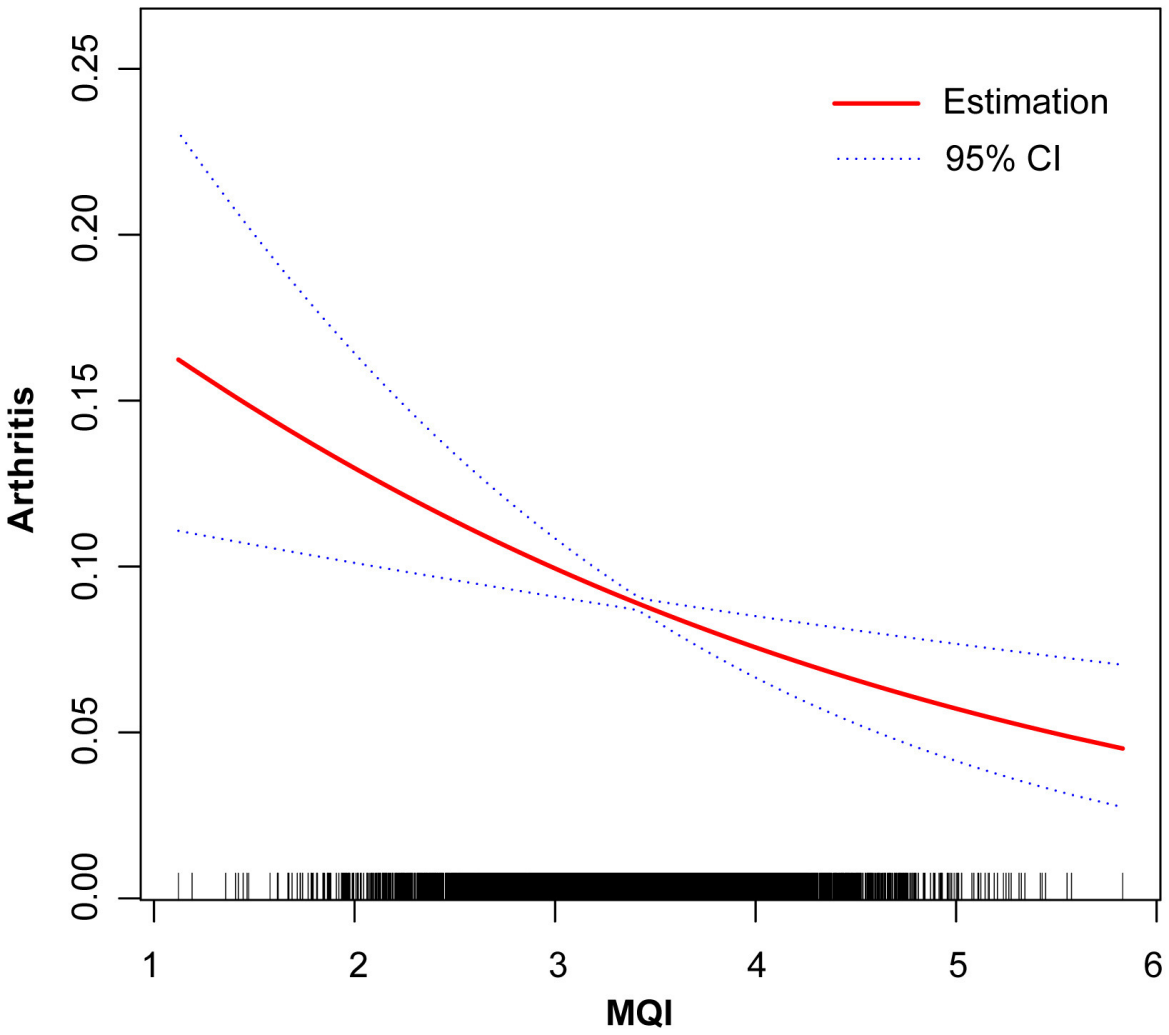
Linear relationship between Muscle Quality Index (MQI) and Arthritis by the generalized additive model. The X-axis represents MQI, and the Y-axis represents the Arthritis. The blue bands represent the 95% confidence interval for the fitted line, adjusted for age, gender, race, educational level, income to poverty ratio (PRI), marital status, diabetes mellitus, smoking status, drinking status, Vitamin B12, sedentary activity, sleep duration.

**Figure 4:**
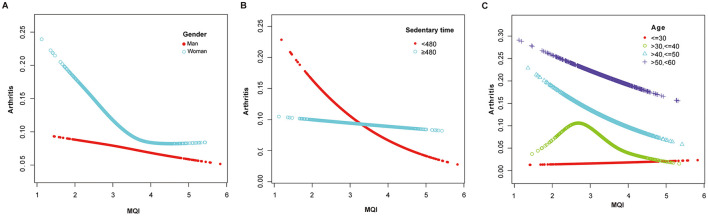
**(A)** Adjusted Dose-Response Relationship Between MQI and Arthritis Incidence Stratified by gender. Age, race, educational level, income to poverty ratio (PRI), marital status, diabetes mellitus, smoking status, drinking status, Vitamin B12, sedentary activity, sleep duration were adjusted. **(B)** Adjusted Dose-Response Relationship Between MQI and Arthritis Incidence Stratified by sedentary time. Age, gender, race, educational level, income to poverty ratio (PRI), marital status, diabetes mellitus, smoking status, drinking status, Vitamin B12, sleep duration were adjusted. **(C)** Adjusted Dose-Response Relationship Between MQI and Arthritis Incidence Stratified by age. Gender, race, educational level, income to poverty ratio (PRI), marital status, diabetes mellitus, smoking status, drinking status, Vitamin B12, sedentary activity, sleep duration were adjusted.

The results of the generalized additive model (GAM) analysis in Figure 3 show that an increase in MQI is associated with a decrease in the prevalence of arthritis, with a particularly significant decrease at the lower end of the MQI range. Table 3 and Figures 4A–C show the stratified effects of muscle mass index (MQI) on arthritis, analyzed by age (30–40 years), sex (female), and non-sedentary population (< 480 min/day). The prevalence of arthritis decreased with increasing MQI in both men and women. The prevalence of arthritis was higher in women than in men at all MQI levels, and the difference was especially pronounced at low MQI. At higher MQI levels, the prevalence in men and women converged. In women, MQI was associated with a reduced prevalence of arthritis (OR = 0.69, *P* = 0.003) with a threshold of 3.75, below which the prevalence of arthritis was significantly lower (OR = 0.55, *P* < 0.001) and above which it was not significant (*P* = 0.241). For the non-sedentary population, the MQI was negatively associated with arthritis (OR = 0.62, *P* < 0.001) with a threshold of 4.24, below which the prevalence of arthritis was significantly lower (OR = 0.55, *P* < 0.001) and above which it was not significant (*P* = 0.131). The MQI was negatively associated with the prevalence of arthritis in people aged 30–40 years (OR = 0.46, *P* < 0.001). The threshold for the MQI was 2.75, below which the prevalence of arthritis was non-significant (*P* = 0.202), whereas above this value, the association was significant and had a protective effect (OR = 0.29, *P* < 0.001). Analyses were adjusted for potential confounders such as demographic, socioeconomic, and lifestyle factors.

**Table 3:** Effect of muscle quality index on arthritis, stratified by age (30,40), gender (female), sedentary time (<480 min).

**Variable**	**OR**	**95% CI**	***P-*value**
**Age (30,40)**			
MQI	0.46	(0.30, 0.71)	< 0.001
**Inflection piont**			
< 2.48	248.00	(0.73, 83,925.32)	0.064
>2.48	0.31	(0.18, 0.52)	< 0.001
*P* for Log-likelihood ratio test			0.002
**Gender (female)**			
MQI	0.69	(0.54, 0.88)	0.003
**Inflection piont**			
< 3.75	0.55	(0.40, 0.76)	< 0.001
>3.75	1.57	(0.74, 3.36)	0.241
P for Log-likelihood ratio test			0.034
**Sedentary time (**<**480 min)**			
MQI	0.62	(0.48, 0.80)	< 0.001
**Inflection piont**			
< 4.24	0.55	(0.42, 0.72)	< 0.001
>4.24	2.88	(0.73, 11.37)	0.131
*P* for Log-likelihood ratio test			0.048

Age, Gender, Race, Educational level, Marital status, Diabetes mellitus, Smoking history, Drinking status, Sedentary activity, Vitamin B12 and Sleep duration were adjusted.

OR, Odds ratio.

95% CI, 95% confdence interval.

## 4 Discussion

This study, based on NHANES 2011–2014 data, found that MQI was significantly and negatively associated with arthritis prevalence. Each unit increase in MQI was associated with a 38% reduction in arthritis prevalence when not adjusted for covariates; the correlation remained significant after adjusting for covariates such as age, gender, and race, as well as further adjusting for confounders. Subgroup analyses showed that higher MQI was significantly associated with a lower prevalence of arthritis among people aged 30 to 50 years, women, and non-hispanic black people, suggesting a potential benefit of higher MQI for arthritis prevention.

To our knowledge, this is the first study to explore the correlation between MQI and arthritis. Previous studies have extensively explored the relationship between muscle mass and arthritis. Guede-Rojas et al. (18) on the other hand, noted that higher muscle mass was significantly associated with improved cardiometabolic status in patients with osteoarthritis of the hip and knee joints. Peng et al. (19) based on the 1999–2006 NHANES data, found that lower muscle mass was associated with an increased prevalence of arthritis increased prevalence of arthritis. Lee et al. (20) showed that lower leg muscle mass was significantly associated with an increased prevalence of knee osteoarthritis, suggesting that leg muscles have a significant impact on joint stability. Wang et al. (21) further showed that the combined effect of low muscle mass and obesity may significantly increase the prevalence of osteoarthritis. Jeon et al.'s (22) study, on the other hand, found that low skeletal muscle mass was significantly associated with significant association with imaging degradation of osteoarthritis in the knee but to a lesser extent in the hip and spine. In addition, Suh et al. (23) found that body composition was strongly associated with the development of knee osteoarthritis in women, whereas relatively few studies have been conducted in the male population. Jeong and Lee (24) found that low socioeconomic status was associated with a high prevalence of osteoarthritis, with low-income and low-educated individuals bearing a greater burden of disease. Driban et al. also emphasized that ignoring comorbidities and lifestyle in studies and other factors can lead to biased results (25), as lifestyle, such as physical activity, is critical for muscle mass and osteoarthritis progression (26). In addition, limited analyses across race, gender, and age groups exacerbate the complexity of understanding. Fu et al. (27) noted differences in the functional impact of osteoarthritis across genders, calling for more nuanced analyses.

Decreased muscle mass may influence the development and progression of arthritis through multiple biological pathways. Muscle tissue plays a vital role in maintaining joint stability, and inadequate muscle mass can result in joint instability and increased mechanical stress, potentially accelerating cartilage degradation (28, 29). This is particularly relevant among older adults, where reduced muscle strength is a known contributor to joint overload and the onset of arthritis. Baker et al. (30) found that reduced muscle mass and density were significantly associated with joint damage in patients with rheumatoid arthritis. Furthermore, systemic inflammation, often elevated in individuals with low muscle mass, may mediate the progression of arthritis. Wang et al. (31) reported increased levels of TNF-α and IL-6 in arthritis patients, both of which are also elevated in states of muscle atrophy. These inflammatory cytokines can activate catabolic signaling pathways such as NF-κB, promoting further degradation of both cartilage and muscle tissue (32). These findings support the hypothesis that improving muscle quality may serve as a preventive strategy for arthritis. Nonetheless, due to the cross-sectional design of our study, a causal relationship cannot be definitively established.

Conversely, arthritis may itself contribute to decreased muscle quality. Joint pain, stiffness, andlimited mobility associated with arthritis often lead to reduced physical activity and muscle disuse, resulting in atrophy over time. Chronic systemic inflammation may also impair protein synthesis in muscle cells, further contributing to a decline in muscle quality (33). As such, the observed inverse association may reflect a bidirectional relationship. It is also worth noting that the biological mechanisms underlying arthritis can vary between subtypes. For instance, rheumatoid arthritis is characterized by autoimmune-mediated inflammation, whereas osteoarthritis involves degenerative and biomechanical factors (4, 5). These distinct pathophysiological processes may influence how muscle quality interacts with arthritis risk and progression.

In addition, the role of vitamins in arthritis has been the subject of research, especially their potential impact on the inflammatory process and joint health. Vitamin B12 is essential for various metabolic functions, including DNA synthesis and the metabolism of homocysteine, an amino acid implicated in inflammation and cardiovascular disease (34). Vitamin B12 deficiency leads to elevated homocysteine levels, which are associated with increased inflammation and may exacerbate diseases such as arthritis, while inflammatory processes related to arthritis may impair vitamin B12 absorption and utilization, creating a vicious cycle of deficiency and symptom worsening (35).

Using subgroup analyses (Figure 2), we found a significant effect of age on the association between muscle mass index (MQI) and arthritis. A one-unit increase in MQI was significantly associated with a 53 and 34% reduction in the prevalence of arthritis in the 30–40 and 40–50-year-olds, respectively, but was not statistically significant in the under 30 and over 50-year-olds. This may be explained by the fact that the effect of MQI on arthritis was not significant in the younger age group due to the lower prevalence of arthritis and generally better muscle mass, whereas in the middle-aged population, the protective effect of MQI against arthritis was more pronounced with increasing age and decreasing muscle mass; however, in the population older than 50 years of age, the effect of MQI on arthritis may be increased due to the impact of other risk factors, such as the burden of chronic disease and bone deterioration, resulting in a greater association between MQI and arthritis. Increased influence on arthritis, resulting in a weakened relationship between MQI and arthritis. There is a significant negative correlation between age and MQI, with individuals tending to deteriorate their muscle mass as they age. A study by Nascimento et al. (36) emphasized that MQI can be effectively predicted by a variety of factors, including age, with a significant difference between eighty-somethings and younger older adults. In addition, the prevalence and severity of various types of arthritis tend to increase with age, with the onset of arthritis usually occurring in middle age or later, with the average age of onset reported to be around 40 years old. Reyes et al. found that the average age of onset of arthritis in individuals with a high BMI and an increased risk for all types of arthritis was around 48 years old, which suggests that the onset of the disease may occur in the late forties (6).

This study has significant strengths and limitations. First, the data were obtained from the representative and authoritative NHANES national database with a wide sample, ensuring the external validity of the results. Second, after adjusting for multiple covariates, the negative correlation between MQI and arthritis prevalence remained significant, demonstrating the robustness of the results. In addition, subgroup analyses revealed more significant associations in specific populations, providing a scientific basis for individualized interventions. However, there are limitations to this study. As a cross-sectional study, it was not possible to determine a causal relationship between MQI and arthritis. Second, despite adjusting for multiple covariates, there may have been uncontrolled confounders. Finally, the study was based on self-reported arthritis diagnostic data, which may be subject to reporting bias and affect the accuracy of the results. Further, mechanistic differences in arthritis type may have influenced the results, but due to insufficient data, the sample size was indeed too large for categorical analyses. Thus, this study is a preliminary exploration of general trends, and further studies are needed to validate the categorical effects in subsequent studies.

## 5 Conclusion

In conclusion, we found that muscle mass index (MQI) was significantly and negatively associated with the prevalence of arthritis, and the association was particularly significant in women in the 30–40 age group and in individuals with less sedentary time. The analysis showed that the association between MQI and arthritis was characterized by non-linearity, with inflection point effects in some subgroups. The results of the present study remained robust after adjusting for multiple confounders, emphasizing that improving muscle mass may be an important strategy for arthritis prevention. However, due to the limitations of the cross-sectional design, future longitudinal studies are needed to validate the causal relationship between MQI and arthritis and its underlying mechanisms further.

## Data Availability

The raw data supporting the conclusions of this article will be made available by the authors, without undue reservation.

## References

[1] Global Burden of Disease Collaborators. Global burden of 369 diseases and injuries in 204 countries and territories, 1990-2019: a systematic analysis for the Global Burden of Disease Study 2019. Lancet. (2020) 396:1204–1222. 10.1016/s0140-6736(20)30925-9 33069326 PMC7567026

[2] Fallon EA Boring MA Foster AL Stowe EW Lites TD Odom EL. Prevalence of diagnosed arthritis—United States, 2019-2021. MMWR Morb Mortal Wkly Rep. (2023) 72:1101–7. 10.15585/mmwr.mm7241a1 37824422 PMC10578950

[3] Duong V Oo WM Ding C Culvenor AG Hunter DJ. Evaluation and treatment of knee pain: a review. JAMA. (2023) 330:1568–80. 10.1001/jama.2023.19675 37874571

[4] Wang F Palmer N Fox K Liao KP Yu KH Kou SC. Large-scale real-world data analyses of cancer risks among patients with rheumatoid arthritis. Int J Cancer. (2023) 153:1139–50. 10.1002/ijc.34606 37246892 PMC10524922

[5] Hunter DJ Bierma-Zeinstra S. Osteoarthritis. Lancet. (2019) 393:1745–59. 10.1016/S0140-6736(19)30417-9 31034380

[6] Reyes C Garcia-Gil M Elorza JM Mendez-Boo L Hermosilla E Javaid MK. Socio-economic status and the risk of developing hand, hip or knee osteoarthritis: a region-wide ecological study. Osteoarthritis Cartilage. (2015) 23:1323–1329. 10.1016/j.joca.2015.03.020 25819582

[7] Janssen I Heymsfield SB Ross R. Low relative skeletal muscle mass (sarcopenia) in older persons is associated with functional impairment and physical disability. J Am Geriatr Soc. (2002) 50:889–896. 10.1046/j.1532-5415.2002.50216.x 12028177

[8] Landi F Liperoti R Fusco D Mastropaolo S Quattrociocchi D Proia A. Sarcopenia and mortality among older nursing home residents. J Am Med Dir Assoc. (2012) 13:121–6. 10.1016/j.jamda.2011.07.004 21856243

[9] Sayer AA Robinson SM Patel HP Shavlakadze T Cooper C Grounds MD. New horizons in the pathogenesis, diagnosis and management of sarcopenia. Age Ageing. (2013) 42:145–150. 10.1093/ageing/afs191 23315797 PMC3575121

[10] Newman AB Kupelian V Visser M Simonsick EM Goodpaster BH Kritchevsky SB. Strength, but not muscle mass, is associated with mortality in the health, aging and body composition study cohort. J Gerontol A Biol Sci Med Sci. (2006) 61:72–77. 10.1093/gerona/61.1.72 16456196

[11] Weng L Xu Z Chen Y Chen C. Associations between the muscle quality index and adult lung functions from NHANES 2011-2012. Front Public Health. (2023) 11:1146456. 10.3389/fpubh.2023.1146456 37234758 PMC10206396

[12] You Y Chen Y Zhang Q Yan N Ning Y Cao Q. Muscle quality index is associated with trouble sleeping: a cross-sectional population based study. BMC Public Health. (2023) 23:489. 10.1186/s12889-023-15411-6 36918831 PMC10012435

[13] Chen Y Lin W Fu L Liu H Jin S Ye X. Muscle quality index and cardiovascular disease among US population-findings from NHANES 2011-2014. BMC Public Health. (2023) 23:2388. 10.1186/s12889-023-17303-1 38041010 PMC10691039

[14] Caamaño-Navarrete F Jerez-Mayorga D Alvarez C Del-Cuerpo I Cresp-Barría M Delgado-Floody P. Muscle quality index in morbidly obesity patients related to metabolic syndrome markers and cardiorespiratory fitness. Nutrients. (2023) 15:2458. 10.3390/nu15112458 37299421 PMC10254905

[15] Barahona-Fuentes G Huerta Ojeda Á Romero GL Delgado-Floody P Jerez-Mayorga D Yeomans-Cabrera MM. Muscle Quality Index is inversely associated with psychosocial variables among Chilean adolescents. BMC Public Health. (2023) 23:2104. 10.1186/s12889-023-16978-w 37884950 PMC10601194

[16] Baah E Kohlmeier M. Obesity and the development of arthritis among adults in the United States using NHANES data. Clin Med Insights Arthritis Musculoskelet Disord. (2024) 17:11795441241264820. 10.1177/11795441241264820 39091588 PMC11292711

[17] Wen Z Gu J Chen R Wang Q Ding N Meng L. Handgrip strength and muscle quality: results from the national health and nutrition examination survey database. J Clin Med. (2023) 12:3184. 10.3390/jcm12093184 37176623 PMC10179381

[18] Guede-Rojas F Ibacache-Saavedra P Leal MI Tuesta M Durán-Marín C Carrasco-Marín F. A Higher skeletal muscle mass and lower adiposity phenotype is associated with better cardiometabolic control in adults with hip and knee osteoarthritis: results from the Chilean national health survey 2016-2017. Nutrients. (2023) 15:4263. 10.3390/nu15194263 37836547 PMC10574707

[19] Peng P Wu J Fang W Tian J He M Xiao F. Association between sarcopenia and osteoarthritis among the US adults: a cross-sectional study. Sci Rep. (2024) 14:296. 10.1038/s41598-023-50528-z 38167445 PMC10761973

[20] Lee SY Ro HJ Chung SG Kang SH Seo KM Kim DK. Low Skeletal muscle mass in the lower limbs is independently associated to knee osteoarthritis. PLoS One. (2016) 11:e0166385. 10.1371/journal.pone.0166385 27832208 PMC5104343

[21] Wang X Xie L Yang S. Association between weight-adjusted-waist index and the prevalence of rheumatoid arthritis and osteoarthritis: a population-based study. BMC Musculoskelet Disord. (2023) 24:595. 10.1186/s12891-023-06717-y 37474953 PMC10357613

[22] Jeon H Lee SU Lim JY Chung SG Lee SJ Lee SY. Low skeletal muscle mass and radiographic osteoarthritis in knee, hip, and lumbar spine: a cross-sectional study. Aging Clin Exp Res. (2019) 31:1557–62. 10.1007/s40520-018-1108-5 30617856

[23] Suh DH Han KD Hong JY Park JH Bae JH Moon YW. Body composition is more closely related to the development of knee osteoarthritis in women than men: a cross-sectional study using the Fifth Korea National Health and Nutrition Examination Survey (KNHANES V-1, 2). Osteoarthritis Cartilage. (2016) 24:605–11. 10.1016/j.joca.2015.10.011 26518994

[24] Jeong KY Lee HJ. Prevalence of Knee Osteoarthritis and health-related quality of life in stroke patients over 60 years old: a cross-sectional study using Korean National Health and Nutrition Examination Survey V. Ann Geriatr Med Res. (2021) 25:178–186. 10.4235/agmr.21.0053 34275255 PMC8497948

[25] Driban JB Eaton CB Amin M Stout AC Price LL Lu B. Glucose homeostasis influences the risk of incident knee osteoarthritis: Data from the osteoarthritis initiative. J Orthop Res. (2017) 35:2282–2287. 10.1002/jor.23531 28128478 PMC5529273

[26] Gustafsson K Kvist J Eriksson M Dahlberg LE Rolfson O. Socioeconomic status of patients in a Swedish national self-management program for osteoarthritis compared with the general population-a descriptive observational study. BMC Musculoskelet Disord. (2020) 21:10. 10.1186/s12891-019-3016-z 31906904 PMC6945568

[27] Liu X Huang Y Fu J. Associations of arthritis with functional disability and depressive symptoms in general US adults: NHANES 1988-1994 and 1999-2018. Aging Med (Milton). (2024) 7:705–16. 10.1002/agm2.12379 39777093 PMC11702379

[28] Peshkova M Lychagin A Lipina M Matteo BD Anzillotti G Ronzoni F. Gender-related aspects in osteoarthritis development and progression: a review. Int J Mol Sci. (2022) 23:2767. 10.3390/ijms23052767 35269906 PMC8911252

[29] Verhoeven F Tordi N Prati C Demougeot C Mougin F Wendling D. Physical activity in patients with rheumatoid arthritis. Joint Bone Spine. (2016) 83:265–270. 10.1016/j.jbspin.2015.10.002 26639220

[30] Baker JF Von Feldt J Mostoufi-Moab S Noaiseh G Taratutu E Kim W. Deficits in muscle mass, muscle density, and modified associations with fat in rheumatoid arthritis. Arthritis Care Res. (2014) 66:1612–1618. 10.1002/acr.22328 24664868 PMC4551488

[31] Wang L Song G Zheng Y Tan W Pan J Zhao Y. Expression of semaphorin 4A and its potential role in rheumatoid arthritis. Arthritis Res Ther. (2015) 17:227. 10.1186/s13075-015-0734-y 26303122 PMC4549119

[32] Shadfar S Couch M McKinney K Weinstein LJ Yin X Rodríguez JE. Oral resveratrol therapy inhibits cancer-induced skeletal muscle and cardiac atrophy *in vivo*. Nutr Cancer. (2011) 63:749–762. 10.1080/01635581.2011.563032 21660860 PMC3623008

[33] Cronström A Häger CK Thorborg K Ageberg E. Factors associated with sports function and psychological readiness to return to sports at 12 months after anterior cruciate ligament reconstruction: a cross-sectional study. Am J Sports Med. (2023) 51:3112–3120. 10.1177/03635465231192983 37681565 PMC10543957

[34] Lopes SC Gadelha DD Carvalho MMDd Fernandes VO Montenegro Jr RM. Vitamin B12 deficiency: metabolic effects, clinical evaluation, and treatment. (2019) 59:40. 10.20513/2447-6595.2019v59n2p40-49

[35] Porter K Hoey L Hughes CF Ward M McNulty H. Causes, consequences and public health implications of low B-vitamin status in ageing. Nutrients. (2016) 8:725. 10.3390/nu8110725 27854316 PMC5133110

[36] Nascimento DDC Prestes J de Sousa Diniz J Beal PR Alves VP Stone W. Comparison of field- and laboratory-based estimates of muscle quality index between octogenarians and young older adults: an observational study. J Exerc Rehabil. (2020) 16:458–66. 10.12965/jer.2040668.334 33178648 PMC7609849

